# Comparison of adverse events between intravitreal anti-VEGF and laser photocoagulation for treatment-requiring retinopathy of prematurity: a systematic review

**DOI:** 10.1007/s10792-022-02480-6

**Published:** 2022-10-10

**Authors:** Georgios N. Tsiropoulos, Aikaterini K. Seliniotaki, Anna-Bettina Haidich, Nikolaos Ziakas, Asimina Mataftsi

**Affiliations:** 1 2nd Department of Ophthalmology, School of Medicine, Faculty of Health Sciences, Aristotle University of Thessaloniki, Thessaloniki, Greece; 2 Department of Hygiene, Social-Preventive Medicine and Medical Statistics, School of Medicine, Faculty of Health Sciences, Aristotle University of Thessaloniki, Thessaloniki, Greece

**Keywords:** Retinopathy of prematurity, Anti-vascular endothelial growth factor, Laser photocoagulation, Adverse events, Complications

## Abstract

**Purpose:**

To synthesize existing evidence on adverse events, complications, and unfavorable outcomes of current treatment modalities for treatment-requiring retinopathy of prematurity (TR-ROP).

**Methods:**

PubMed, Cochrane Central Register of Controlled Trials, Scopus, EMBASE, Trip Database, and the gray literature available were searched. Randomized Clinical Trials and observational studies comparing the adverse events of intravitreal anti-VEGF injections (bevacizumab, ranibizumab, aflibercept, pegaptanib, conbercept) and laser photocoagulation (LPC) as treatment modalities for infants with TR-ROP were included. The main outcomes compared between the two treatment modalities were: 1. Refractive Errors and Biometry Parameters, 2. Adverse events, complications, and unfavorable outcomes, 3. Disease Recurrence/Disease Regression/Need for retreatment, 4. Neurodevelopmental Outcomes.

**Results:**

Higher quality studies concluded that LPC leads to greater rates of myopia than intravitreal anti-VEGF treatment while the rate of adverse events and of unfavorable neurodevelopmental outcomes is similar. However, there was controversy among the included studies concerning the rate of ROP recurrence between intravitreal anti-VEGF injections and LPC.

**Conclusion:**

There is need for future primary studies assessing the adverse events of intravitreal anti-VEGF injections compared with LPC as treatment modalities for infants with TR-ROP.

## Introduction

Retinopathy of prematurity (ROP), the leading cause of infants’ blindness all over the world [1], is a disorder of the retinal vasculature, with pathologic vessels growing into the vitreous instead of the retina [2]. Before introducing the use of anti-vascular endothelial growth factor (anti-VEGF) agents in infants, clinical trials had confirmed the efficacy and safety of ablating the avascular peripheral retina to achieve the regression of preretinal neovascularization and prevent ensuing fibrovascular retinal detachments [2]. Treatment-warranted ROP was defined as a set of characteristics that resulted in a 50% chance of an unfavorable outcome, and cryotherapy was the first treatment modality that proved to lower that risk when compared to no treatment. [3]. Subsequently, ET-ROP study showed the efficacy of the laser treatment for a less severe form of treatment-warranting ROP (type 1 ROP), in which the risk of an unwanted outcome was approximately 15% [4].

The BEAT-ROP study [5] was the first prospective, multicenter, stratified, randomized controlled trial (RCT) that attempted a comparison of efficacy between intravitreal bevacizumab (IVB) monotherapy and conventional laser photocoagulation (LPC) for zone 1 or zone 2 posterior stage 3 + ROP. The study reached the conclusion that IVB was beneficial for infants with stage 3 + ROP for zone 1(*P* = 0.003), but not for zone 2 disease (*P* = 0.270). Although BEAT-ROP encouraged many clinicians worldwide to use IVB as first-line treatment for ROP, IVB still remains an off-label modality. A second RCT evaluating ranibizumab followed: RAINBOW study [6], an open-label, multicenter, randomized, three-arm, parallel group, superiority trial, assigned infants with treatment-requiring ROP (TR-ROP) in three groups of 0.2 mg intravitreal ranibizumab (IVR), 0.1 mg IVR and LPC, concluding that ranibizumab 0.2 mg is superior to LPC, while having less unfavorable ocular outcomes. In September 2019, the European Medicines Agency (EMA) approved ranibizumab 0.2 mg as an on-label treatment for infants with TR-ROP. The RAINBOW study provided evidence on the drug’s efficacy as well as on short-term safety issues [6]. However, more information is needed concerning the adverse events (AEs), complications, and unfavorable functional and structural outcomes of the two treatment modalities in the long term.

The aim of this systematic review was to capture the current knowledge regarding the adverse events, the complications and the unfavorable structural and functional outcomes of intravitreal anti-VEGF agents and LPC as treatment modalities for TR-ROP, so as to guide clinical ophthalmologists in their choice of the preferred treatment modality for each case of TR-ROP.

## Methods

### Study characteristics

This study is a systematic review of RCTs and observational studies that compared intravitreal anti-VEGF injections and LPC as treatment modalities in infants with TR-ROP, in terms of adverse events, complications, and unfavorable structural and functional outcomes. Literature search was carried out until 25/7/2020 without restrictions. The study had been registered to PROSPERO with the following registration number: CRD42020189408.

### Eligibility criteria

#### Inclusion criteria

- The included studies were either RCTs or observational studies.
- The participants of each study were infants with ROP that required treatment.
- The included studies evaluated one of the following intravitreal anti-VEGF agents as monotherapy: ranibizumab, bevacizumab, aflibercept, pegaptanib, conbercept, and compared its adverse events, complications, or unfavorable outcomes with one of the following types of LPC, also used as a monotherapy: diode laser, argon laser, Yttrium aluminum garnet (YAG) laser.

#### Exclusion criteria

- The studies that did not involve humans as subjects.
- The studies that reported early-stage outcomes that are also reported in the complete version of the study. In this case, the most complete version was included to avoid duplication of our results.
- The studies that reported the outcome of the interventions conducted to treat an adverse event of a treatment modality for ROP, rather than comparing the outcomes of intravitreal anti-VEGF as monotherapy with LPC, also used as a monotherapy, as treatment modalities for ROP.

### Study outcomes

The outcomes of interest were the comparison of adverse events, complications, and unfavorable structural and functional outcomes between intravitreal anti-VEGF treatment and LPC, and were categorized as follows:

1. *Refractive Errors and Biometry Parameters* This subsection evaluated refractive spherical power, spherical equivalent (SE), cylinder power, best-corrected visual acuity (BCVA), rates of myopia and high myopia, rates of anisometropia, rates of astigmatism and biometric results (e.g., anterior chamber depth (ACD), lens thickness (LT), axial length (AL), and central choroidal thickness (CCT)).
2. *Adverse events, complications, and unfavorable outcomes* This subsection evaluated both ocular and systemic unfavorable outcomes: rates of retinal detachment, vitreous hemorrhage, macular dragging, retinal fold, macular ectopia, endophthalmitis, ocular inflammations, cataract formation, glaucoma, corneal opacity requiring transplantation, and death.
3. *Disease Recurrence/Disease Regression/Need for retreatment* There is not a universal definition of ROP recurrence and its difference from treatment failure. Therefore, the definition of ROP recurrence, treatment failure, or treatment success that each study used, are provided when the respective results are reported.
4. *Neurodevelopmental Outcomes* Reported Bayley-3 scores of cognition, language and motor composite were assessed.
5. *Optical coherence tomography (OCT) and optical coherence tomography angiography (OCTA) Measurements* OCT assessment of the posterior part of the eye, including inner foveal thickness (IFT), outer foveal thickness (OFT), subfoveal choroid thickness (CT), foveal avascular zone (FAZ), foveal vessel density (VD), parafoveal VD, perifoveal VD and macular volume, is presented.
6. *Other reported outcomes* Comparison of serum-free VEGF levels, serum insulin-like growth factor–1 (IGF-1) levels and cardiovascular assessment between infants with TR-ROP that were treated with either intravitreal anti-VEGF or LPC, are included in this subsection.

### Searches and search strategy

**Fig. 1:**
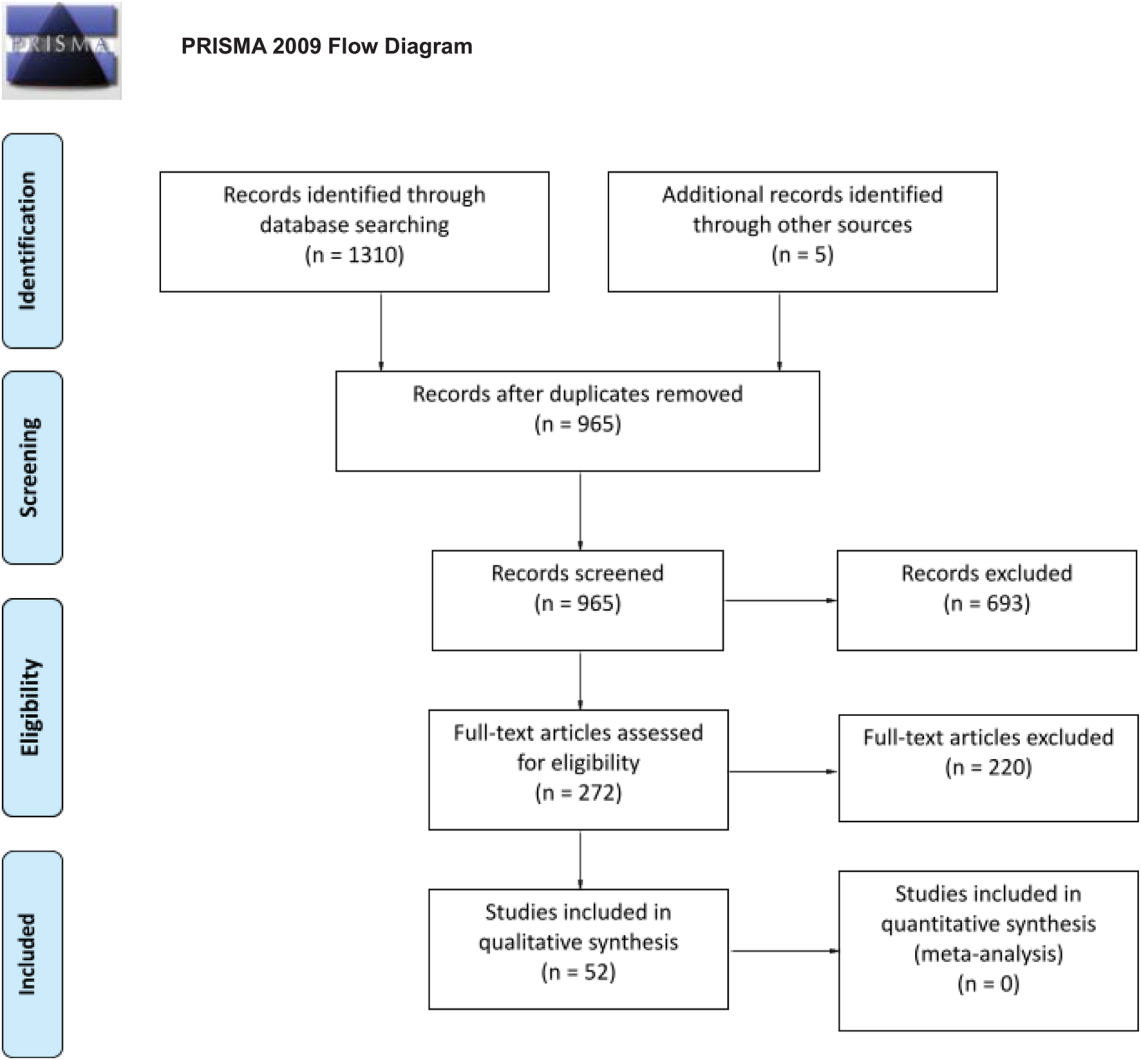
The PRISMA flow diagram of this systematic review. (from: Moher D, Liberati A, Tetzlaff J, Altman DG, The PRISMA Group (2009) Preferred Reporting Items for Systematic Reviews and Meta-Analyses: The PRISMA Statement. PLoS Med 6: e1000097. https://doi.org/10.1371/journal.pmed1000097)

The following databases were searched: PubMed, Cochrane Central Register of Controlled Trials (CENTRAL), Scopus, EMBASE and Trip Database. A search in the gray literature, such as ClinicalTrials.gov and in available conference proceedings of American Academy of Ophthalmology (AAO), European Paediatric Ophthalmological Society (EPOS), American Association for Pediatric Ophthalmology and Strabismus (AAPOS) and EURETINA, has also been conducted. Furthermore, to ensure a systematic search of the existing literature, reference lists of any included study were scanned rigorously to find eligible studies that the search may had missed. The basic search terms that corresponded to each element of the research question were used for the search strategies in all databases. Every step of the systematic review process was performed by two independent researchers. In the case of disagreements, the final decision was determined by the senior author. After duplicates were removed, all studies were searched by title and abstract. Studies that did not satisfy the research question were excluded. Full-text screening was performed in the remaining, potentially eligible studies. Whenever a study had been published in different versions, the latest and most complete version was selected. The PRISMA flow diagram is presented in Fig. 1 [7].

### Risk-of-bias (quality) assessment

The risk-of-bias (quality) assessment for the RCTs and non-randomized studies of interventions (NRSI) was conducted with the use of the RoB 2.0 [8] and ROBINS-I [9] tools, respectively.

### Patient consent form

No patient consent forms were needed as this is a systematic review.

## Results

### Refractive errors and biometry

#### Comparison between three groups (IVB, IVR and diode LPC)

The observational studies that compared the refractive errors and biometric measurements between IVB, IVR and diode LPC groups reported no differences between the three groups [10–12], as it is shown in Table 1.

**Table 1:** Observational studies comparing refractive errors and biometric measurements between IVB, IVR, diode LPC treatment modalities for TR-ROP

Authors/first author	Year	Participants	Treatment modalities being compared	Outcomes	Risk-of-bias assessment with ROBINS-I tool^9^
Kang et al. [10]	2019	30 eyes (diode LPC group) 20 eyes (IVB group) 2 eyes (IVR group)	IVB (0.625 mg/0.025 mL), IVR (0.2 mg/0.02 mL), Diode LPC	No differences between groups at age of 4 years in: Mean spherical power (*P* = 0.490, ANOVA test), Mean cylinder power (*P* = 0.290, ANOVA test), Mean spherical equivalence (*P* = 0.600, ANOVA test)	Moderate overall risk of bias
Kabatas et al. [11]	2017	24 eyes of 12 type 1 ROP infants (IVB group), 12 eyes of 6 type 1 ROP infants (IVR group), 72 eyes of 36 type 1 ROP infants (diode LPC group)	IVB (0.625 mg/0.025 mL, 100 mg/ 4 ml flacon) with diode LPC, IVR (0.25 mg/0.025 mL, 10 mg/1 ml flacon) with diode LPC, IVB (0.625 mg/0.025 mL, 100 mg/4 ml flacon) with IVR (0.25 mg/0.025 mL, 10 mg/1 ml flacon)	No differences between groups in Mean spherical equivalent at 18 months CA (*P* = 1.000, *P* = 1.000,* P* = 1.000 respectively), Mean cylindrical value at 18 months CA (*P* = 0.456, *P* = 1.000, *P* = 1.000 respectively)	Serious overall risk of bias due to baseline and time-varying confounding
Gunay et al. [12]	2017	55 infants with TR-ROP (IVB group) 22 infants with TR-ROP (IVR group) 57 infants with TR-ROP (diode LPC group)	IVB (0.625 mg/0.025 mL), IVR (0.25 mg/0.025 mL), Diode LPC	No differences between groups in: Spherical equivalent at 1.5 years CA (*P* = 0.130, ANOVA test), Rate of myopia (SE ≤ − 0.25 D) at 1.5 years CA (*P* = 0.080, ANOVA test), Rate of high myopia (SE ≤ − 5.0 D) at 1.5 years CA (*P* = 0.970, ANOVA test) Mean AL at 1.5 years CA (*P* = 0.390, ANOVA test)	Critical overall risk of bias due to baseline and time-varying confounding

*IVB* intravitreal bevacizumab, *IVR* intravitreal ranibizumab, *LPC* laser photocoagulation, *TR-ROP* treatment-requiring retinopathy of prematurity, *ROBINS-I* risk of bias in non-randomized studies of interventions, *ROP* retinopathy of prematurity, *ANOVA* analysis of variance, *SE* spherical equivalent, *D* diopter(s), *CA* corrected age, *AL* axial length (mm)

#### Comparison between 2 groups

All RCTs [13, 14] and observational studies [15–36] that compared intravitreal anti-VEGF injections with LPC for refractive errors and biometry are shown in Table 2. Raghuram et al. [15] reported a more myopic median refraction and a greater rate of myopia ≤ − 0.25 diopters (*D*) in diode LPC-treated eyes than in IVB-treated eyes at 18–24 months of age (*P* = 0.020, *P* = 0.040, respectively). These outcomes are at moderate overall risk of bias and of the highest quality among the observational studies.

**Table 2:** Randomized clinical trials and observational studies comparing refractive errors and biometric measurements between anti-VEGF and LPC treatment modalities for TR-ROP

Authors/first author	Study Type	Year	Participants	Treatment modalities being compared	Outcomes	Risk-of-bias assessment with RoB 2 tool^8^ or with ROBINS-I tool^9^
Roohipoor et al. [13]	Randomized clinical trial	2019	232 eyes of 116 infants with type 1 ROP in zone 2 (stage 2 or 3 ROP with plus disease)	IVB (0.625 mg/0.025 ml), Diode LPC	No differences between groups in: Mean spherical power at 90 weeks PMA (*P* = 0.360) Mean cylindrical power at 90 weeks PMA (*P* = 0.430)	Some Concerns
Geloneck et al. [14]	Randomized clinical trial	2014	211 eyes of 109 infants with zone 1 or zone 2 posterior ROP with stage 3 + ROP or aggressive posterior ROP	IVB (0.625 mg/0.025 ml), Diode LPC	Eyes in LPC group had: More myopic spherical equivalent at 2.5 years of age (*P* < 0.001) Greater incidence of very high myopia (≤ − 8.00 D) at 2.5 years of age (*P* < 0.001)	Low
Raghuram et al. [15]	Observational study	2019	60 eyes of 34 infants with TR-ROP	IVB (0.625 mg/0.025 ml), Diode LPC	Eyes in diode LPC group had: More myopic median refraction at 18–24 months of age (*P* = 0.020) Greater rate of myopia ≤ − 0.25 D at 18–24 months of age (*P* = 0.040) No differences between groups in: Median visual acuity at 18–24 months of age (*P* = 0.850) Rate of myopia ≤ − 5.00 D at 18–24 months of age (*P* = 0.360)	Moderate overall risk of bias
Chen et al. [16]	Observational study	2019	47 eyes of 25 infants with type 1 ROP	IVB (0.625 mg/0.025 ml), Diode LPC	Eyes in diode LPC group had: Greater mean spherical equivalent (*P* = 0.010) Greater average keratometry (*P* = 0.010) Greater mean LT (*P* = 0.020) Lower mean ACD (*P* = 0.010) No differences between groups in: BCVA (*P* = 0.180) Cylinder power (*P* = 0.060) Mean AL (*P* = 0.580)	Critical overall risk of bias due to selection bias
Therani et al. [17]	Observational study	2019	111 eyes of 64 infants with TR-ROP	IVB (dose unspecified), LPC (type unspecified)	Eyes in LPC group had: Greater median spherical equivalent at 18–24 months CA (*P* = 0.020) Mean visual acuity at 18–24 months CA (*P* = 0.850)	Serious overall risk of bias
Manuchian et al. [18]	Observational study	2019	44 infants with TR-ROP	IVB (dose unspecified), LPC (type unspecified)	Eyes in LPC group had: Greater rate of cumulative insults to binocularity (*P* = 0.040) No difference between the two groups in Rate of binocularity between the groups (*P* = 0.270)	Critical overall risk of bias due to baseline confounding and missing information concerning selection bias and selective reporting of results
Roohipoor et al. [19]	Observational study	2018	986 eyes of 493 infants with type 1 ROP	IVB (0.625 mg/0.025 ml), Diode LPC	The diode LPC group had Greater mean spherical power (*P* = 0.020) Greater mean spherical equivalent (*P* = 0.020) Greater rate of eyes with high myopia (Spherical Power < − 4.00 D) (*P* = 0.020) No differences were found between the two treatment groups in: Mean cylindrical power (*P* > 0.050)	Moderate overall risk of bias
Lee et al. [20]	Observational study	2018	80 eyes of 42 infants with TR-ROP	IVB (0.625 mg/0.025 ml), Diode LPC	The diode LPC group had Higher visual acuity (logMAR) (*P* < 0.050) More myopic mean spherical power (*P* < 0.050) More myopic mean spherical equivalent (*P* < 0.050) Lower mean ACD (*P* < 0.050) No differences were found between the two treatment groups in: Mean corrected visual acuity (*P* > 0.050) Mean cylindrical power (*P* > 0.050) Axis (with the rule) (*P* > 0.050) Mean K1 (*P* > 0.050) Mean K2 (*P* > 0.050) Mean K1-K2 average (*P* > 0.050) Mean CCT (*P* > 0.050) Mean AL (*P* > 0.050)	Critical overall risk of bias due to selection bias
Mueller et al. [21]	Observational study	2017	54 infants with type 1 ROP	IVB (0.625 mg/0.025 ml), Diode LPC	Diode LPC group had: More myopic spherical equivalent at 12–15 months post-treatment (*P* = 0.001) No differences between groups concerning: Mean visual acuity at 12–15 months post-treatment (*P* = 0.290)	Serious overall risk of bias due to time-varying confounding
Kong et al. [22]	Observational study	2015	80 eyes of 42 infants with TR-ROP	IVB (0.625 mg/0.025 ml), LPC (type unspecified)	LPC group had: Higher rate of eyes with myopia ≤ − 0.25 D (*P* = 0.030 at 1 year of age, *P* = 0.002 at 2 years of age) Higher rate of eyes with myopia ≤ − 5.00 D (*P* = 0.006 at 1 year of age, *P* = 0.001 at 2 years of age) Greater myopic refractive errors (*P* = 0.020 at 1-year of age, *P* = 0.030 at 2 years of age)	Serious overall risk of bias due to time-varying confounding
Vujanović et al. [23]	Observational study	2017	132 eyes of 66 infants with TR-ROP	IVB (0.625 mg/0.025 ml), LPC (type unspecified)	Eyes of LPC group had: Higher rate of emmetropia (*P* < 0.010) Higher rate of high hyperopia (Spherical Equivalent > 4.00 D) (*P* < 0.010) Higher rate of anisometropia (*P* < 0.050) Higher rate of mean ACD (*P* < 0.010) Lower mean LT (*P* < 0.010) No difference between groups in: Rate of myopia Rate of high myopia Rate of hyperopia Rate of astigmatism presence Rates of astigmatism distribution from 1.00 to 2.00 D cylinder, ≥ 2.00 D cylinder Rates of anisometropia ≥ 1.00 D to < 2.00 D, ≥ 2.00 D mean AL	Serious overall risk of bias due to baseline confounding
Lolas et al. [24]	Observational study	2017	144 eyes of 72 patients with posterior zone 2, zone 1 ROP, and aggressive posterior ROP	IVB (0.625 mg/0.025 ml), YAG laser	No difference between groups in: Visual acuity (Teller test) Median spherical equivalent Median cylinder at 10-month follow-up	Serious risk of bias due to baseline confounding
Gunay et al. [25]	Observational study	2016	63 infants with TR-ROP	IVB (0.625 mg/0.025 ml), Diode LPC	Eyes in IVB group had: Lower median LT (*P* = 0.010) at 1-year CA No difference between groups in: Mean spherical equivalent (*P* = 0.370) Median AL (*P* = 0.350) Median ACD (*P* = 0.610) Incidence of myopia (spherical equivalent ≤ − 0.25 D) Incidence of high myopia (spherical equivalent ≤ − 5.00 D) (*P* = 0.080) Incidence of high anisometropia (*P* = 0.200) at 1-year CA	Critical overall risk of bias due to selection bias
Gunay et al. [26]	Observational study	2016	71 eyes of 31 infants with type 1 ROP and aggressive posterior ROP	IVB (100 mg/4 ml flacon), Diode LPC	No difference between groups in: Mean spherical equivalent (*P* = 0.980)	serious risk of bias due to baseline confounding
Gunay et al. [27]	Observational study	2015	78 eyes of 40 infants with aggressive posterior ROP	IVB (0.625 mg/0.025 ml), Diode LPC	Eyes in LPC group had: More myopic mean spherical equivalent at a CA of 2 years (*P* = 0.001) More myopic refraction (*P* = 0.010) Higher incidence of refractive anisometropia (*P* = 0.009)	Serious overall risk of bias due to selection bias
Li et al. [28]	Observational study	2015	41 eyes of 21 patients	IVB (0.625 mg/0.025 ml), LPC (type unspecified)	Eyes in LPC group had: More myopic mean spherical equivalent at 1 year of age (*P* = 0.020) and at 3 years of age (*P* = 0.050) No difference between groups in: Mean visual acuity at 3 years of age (*P* = 0.100)	Critical risk of bias due to missing information in different bias assessment domains
Hwang et al. [29]	Observational study	2015	54 eyes of 28 infants with type 1 ROP	IVB (0.625 mg/0.025 ml), Diode LPC	Eyes in LPC group with zone 2 type 1 ROP had: Greater mean spherical power (*P* = 0.004) Greater mean spherical equivalent (*P* = 0.002) No difference between groups in: Mean cylindrical power (*P* = 0.130 for zone 1 and *P* = 0.190 in zone 2)	Serious overall risk of bias due to baseline confounding
Kuo et al. [30]	Observational study	2015	54 eyes of 54 patients with type 1 ROP	IVB (0.5-mg/0.02 ml), Diode LPC	No difference between groups in: Mean spherical equivalent of right eye (*P* = 1.000) Mean spherical equivalent of both eyes (*P* > 0.050)	Critical overall risk of bias, due to selection bias
Isaac et al. [31]	Observational study	2015	55 eyes of 25 infants with type 1 ROP in zone I (stage 2: 2 LPC, stage 3: 8 IVB, 6 LPC) or zone II posterior (stage 3: 15 IVB, 14 LPC)	IVB (0.625 mg/0.025 ml), Diode LPC	No difference between groups in: Mean monocular visual acuity (*P* = 0.340) Mean spherical equivalent (*P* = 0.330) Prevalence of myopia (*P* = 0.080) Prevalence of high myopia (≤ − 5.00 D) (*P* = 0.270) at mean CA of 11.06 (IVB) and 12.1 months (diode LPC)	Moderate overall risk of bias
Isaac et al. [32]	Observational study	2015	44 eyes of 24 infants with type 1 ROP	IVB (0.625 mg/0.025 ml), Diode LPC	No difference between groups in: Mean spherical equivalent (*P* = 0.130) Mean visual acuity at 2 years CA (*P* = 0.300) Prevalence of myopia (*P* = 0.060) Prevalence of high myopia (≤ − 5.00 D) (*P* = 0.320)	Critical overall risk of bias because of missing information for various bias assessment domains
Warren et al. [33]	Observational study	2015	47 infants with threshold ROP	IVB (dose unspecified), Diode LPC	LPC group had: Greater proportion of patients with refractive errors (*P* < 0.050)	Serious overall risk of bias due to time-varying confounding
Harder et al. [34]	Observational study	2013	23 eyes of 12 infants with threshold ROP in zone 1 or zone 2	IVB (0.375 mg or 0.625 mg), Argon LPC	Eyes in argon LPC group had: Greater mean refractive error (*P* = 0.020) Greater prevalence of moderate myopia (≤ − 5.00 D) (*P* = 0.020) Greater mean refractive astigmatism (*P* = 0.030), at 12 months after birth No difference between groups in: Prevalence of high myopia (≤ − 8.00 D) (*P* = 0.100)	Serious overall risk of bias due to selection bias
Harder et al. [35]	Observational study	2012	32 eyes of 16 infants with ROP threshold disease in posterior zone 2 or zone 1 or for pre-threshold ROP in zone 1	IVB (0.375 mg), Argon LPC	Eyes in argon LPC group had: Greater refractive error in left eyes (*P* = 0.020) No difference between groups in: Refractive error of right eyes (*P* = 0.300) Astigmatism of right eyes (*P* = 0.220) Astigmatism of left eyes (*P* = 0.900)	Serious risk of overall bias due to selection bias
Kang et al. [36]	Observational study	2019	314 eyes from 165 infants with type 1 ROP	IVR (0.25 mg/0.025 mL), Diode LPC	Eyes in diode LPC group had: Greater mean spherical equivalent (*P* = 0.030) at a mean follow-up of 36.3 ± 31.9 months	Serious overall risk of bias due to time-varying confounding

*anti-VEGF* anti-vascular endothelial growth factor, *LPC* laser photocoagulation, *TR-ROP* treatment-requiring retinopathy of prematurity, *RoB 2* version 2 of the Cochrane risk-of-bias tool for randomized trials, *ROBINS-I* risk of bias in non-randomized studies of interventions, *ROP* retinopathy of prematurity, *IVB* intravitreal bevacizumab, *PMA* postmenstrual age, *D* diopter(s), *LT* lens thickness, *ACD* anterior chamber depth (mm), *BCVA* best-corrected visual acuity, *AL* axial length (mm), *CA* corrected age, *logMAR* logarithm of the minimum angle of resolution, *K1* horizontal keratometric reading, *K2* vertical keratometric reading, *CCT* central corneal thickness (μm), *YAG* yttrium aluminum garnet, *IVR* intravitreal ranibizumab

### Adverse events, complications, and unfavorable structural outcomes

#### Comparison between three groups

##### RCTs

In the RAINBOW trial [6], unfavorable structural outcomes, defined as structural abnormalities that have potential effects on visual acuity were found in all three arms: 1 infant in the 0.2 mg IVR arm, 5 in the 0.1 mg IVR arm, and 7 infants in the LPC arm. At 24 weeks after initial treatment, death, serious adverse events (SAEs) and non-serious systemic AEs were similar between the treatment groups as 4 deaths occurred in each group. In the 0.2 mg IVR group, one infant had a moderate cataract formation, while in the 0.1 mg IVR group, one infant developed endophthalmitis in one eye. These outcomes are at low overall risk of bias.

#### Comparison between 2 groups

The results of all the RCTs [5, 6, 13, 37–39], and the observational studies [10, 19, 22, 27, 31, 33, 36, 40–47], that compared the adverse events, complications, and unfavorable outcomes between intravitreal anti-VEGF injections and LPC, are displayed in Table 3.

**Table 3:** Randomized clinical trials and observational studies comparing the adverse events, complications, and unfavorable outcomes between intravitreal anti-VEGF and LPC treatment modalities for TR-ROP

Authors/first author	Study Type	Year	Participants	Treatment modalities being compared	Outcomes	Risk-of-bias assessment with RoB 2 tool^8^ or with ROBINS-I tool^9^
Stahl et al. [6]	Randomized clinical trial	2019	225 infants with zone 1 stage 1+, 2+, 3 or 3+ ROP, or APROP	0.2 mg IVR, 0.1 mg IVR, Diode LPC	Infants with unfavorable structural outcome (retrolental membrane obscuring the view of the posterior pole, substantial temporal retinal vessel dragging causing abnormal structural features or macular ectopia, posterior retinal fold involving the macula, or retinal detachment involving the macula.): 1(0.2 mg IVR group), 5 (0.1 mg IVR group), 7 (LPC group) No differences between groups in: Death Serious AEs Non-serious systemic AEs	Low overall risk of bias (“Low” in RoB 2.0 tool)
Shah et al. [43]	Observational study	2019	398 eyes of 199 infants with APROP	Anti-VEGF (type and dose unspecified), LPC (type unspecified)	LPC group had a: Greater rate of eyes with RD (*P* = 0.002)	Serious overall risk of bias due to baseline and time-varying confounding
Zhang et al. [44]	Observational study	2019	283 infants with TR-ROP	IVB (dose unspecified), LPC (type unspecified)	No differences between groups in rate of RD (*P* = 0.190) Other adverse events (vitreous hemorrhage, corneal opacities, cataract, glaucoma, strabismus)	Serious overall risk of bias because of missing information in two bias assessment domains
Kang et al. [10]	Observational study	2019	52 eyes of 27 infants with TR-ROP	Anti-VEGF (either IVB 0.625 mg/0.025 ml, or IVR 0.2 mg/0.02 mL), Diode LPC	No differences between groups in: Rates of complications (systemic complications, death, strabismus requiring operation) (*P* = 0.160)	Moderate overall risk of bias
Roohipoor et al. [13]	Randomized clinical trial	2019	232 eyes of 116 infants with type 1 zone 2 ROP	IVB(0.625 mg/0.025 ml), Diode LPC	IVB group had: Cataract formation in one eye of 159 injected (0.63%) Diode LPC group had: Retinal fold and traction in 2 eyes (2.6%)	Medium overall risk of bias (“Some Concerns” in RoB 2.0 tool)
Kang et al. [36]	Observational study	2019	314 eyes from 165 infants with type 1 ROP	IVR (0.25 mg/0.025 ml), diode LPC	Diode LPC group had a higher incidence rate of: Retinal detachment (*P* = 0.040) Macular dragging (*P* = 0.040) No differences were found between the groups in incidence rate of: Vitreous hemorrhage (*P* = 0.610) Cataract (*P* = 0.730) Glaucoma (*P* = 0.400) Adverse neurodevelopmental outcomes (*P* = 0.490) Strabismus requiring operation (*P* = 0.630)	Moderate overall risk of bias
Barry et al. [46]	Observational study	2019	222 eyes of 115 infants with type 1 ROP	IVB (0.375 to 0.625 mg per eye), LPC (type unspecified)	LPC group of infants before 36 weeks PMA had a higher rate of: RD (*P* = 0.011) No differences between groups in rate of: RD for the whole cohort (treated before, at or after 36 weeks PMA, *P* = 0.373) Death within 8 weeks after treatment (*P* = 1.000)	Serious overall risk of bias due to baseline confounding, time-varying confounding and selection bias
Lyu et al. [42]	Observational study	2019	27 eyes of 14 infants with type 1 ROP	IVR (0.25 mg/0.025 ml), Diode LPC	IVR group had: Greater rate of eyes with unfavorable outcomes (temporally dragged retina over the nerve, retinal detachment, or fold) (*P* = 0.510, Fisher’s exact test)	Serious overall risk of bias due to baseline and time-varying confounding
Blair et al. [40]	Observational study	2018	36 eyes of 19 infants with APROP	IVB (0.5–0.625 mg/ 0.02–0.025 cc), Diode LPC	Diode LPC group had: Higher rate of eyes with poor structural outcome (*P* = 0.002)	Serious overall risk of bias due to baseline and time-varying confounding
Walz et al. [41]	Observational study	2018	166 infants with TR-ROP	Anti-VEGF (type and dose unspecified), LPC (type unspecified)	No differences between the two treatment groups in: Incidence of systemic complications (*P* > 0.050)	Serious overall risk of bias due to baseline confounding
Lepore et al. [37]	Randomized clinical trial	2018	42 eyes of 21 infants with type 1 zone 1 ROP	IVB (0.5 mg/0.02 ml), Diode LPC	Eyes with complete RD 4 weeks after treatment: 1 (IVB group) vs 0 (LPC group)	High overall risk of bias (“High” in RoB 2.0 tool)
Roohipoor et al. [19]	Observational study	2018	986 eyes of 493 infants with type 1 ROP	IVB (0.625 mg/0.025 ml), Diode LPC	Diode LPC group had a higher rate of: Macular dragging (*P* = 0.020) No differences between groups in rate of: RD (*P* = 0.620)	moderate overall risk of bias
Peyton et al. [47]	Observational study	2018	22 infants with type 1 ROP	IVB (dose unspecified), LPC (type unspecified)	No differences between groups in rate of: Death in the period of 12- and 24-month PTA (*P* = 1.000)	Serious overall risk of bias due to baseline confounding
Zhang et al. [39]	Randomized clinical trial	2017	100 eyes of 50 infants with Zone 2 Stage 2 or 3 ROP with plus disease	IVR (0.3 mg/0.03 ml), Diode LPC	No complications (anterior segment ischemia, pupillary membrane, lens opacity, vitreous hemorrhage, retinal detachment, endophthalmitis) between groups	Medium overall risk of bias (“Some Concerns” in RoB 2.0 tool)
Sukgen et al. [45]	Observational study	2017	31 eyes of 16 infants with stage 4A ROP	IVR (0.25 mg/0.025 ml), Diode LPC	No differences between the two treatment groups in: Mean width of partial RD (*P* = 0.806)	Moderate overall risk of bias
Karkhaneh et al. [38]	Randomized clinical trial	2016	158 eyes of 79 infants with Type 1 Zone 2 ROP	IVB (0.625 mg/0.025 ml), Diode LPC	Absence of complications (death, cataract, endophthalmitis, vitreous hemorrhage, retinal detachment) at 54 weeks PMA in both groups	Medium overall risk of bias (“Some Concerns” in RoB 2.0 tool)
Kong et al. [22]	Observational study	2015	80 eyes of 42 infants with type 1 ROP	IVB (0.625 mg/0.025) ml, LPC (type unspecified)	LPC group had: Higher rate of unfavorable structural outcomes at 1 year of chronological age (*P* = 0.020)	Serious overall risk of bias due to baseline and time-varying confounding
Gunay et al. [27]	Observational study	2015	78 eyes of 40 infants with APROP	IVB (0.625 mg/0.025) ml, Diode LPC	Diode LPC group had: Greater rate of strabismus at 2 years CA (*P* = 0.040)	Serious overall risk of bias due to selection bias
Isaac et al. [31]	Observational study	2015	45 eyes of 25 infants with type 1 ROP	IVB (0.625 mg/0.025) ml, Diode LPC	No differences between the two treatment groups in: Favorable outcomes (*P* = 0.080)	moderate overall risk of bias
Warren et al. [33]	Observational study	2015	47 infants with threshold ROP	IVB (dose unspecified), Diode LPC	No differences between the two treatment groups in: Rate of infants with strabismus requiring surgery (*P* > 0.050)	Serious overall risk of bias due to baseline and time-varying confounding
Mintz-Hittner et al. [5]	Randomized clinical trial	2011	300 eyes of 150 infants with zone 1 or zone 2 posterior stage 3 + ROP	IVB (0.625 mg/0.025 ml), Diode LPC	Eyes with macular dragging: 1 (IVB group) vs 22 (LPC group) Eyes with RD: 2 (IVB group) vs 2 (LPC group) Eyes with corneal opacity requiring corneal transplant: 1 (LPC group) Lens opacity requiring cataract removal: 3 (LPC group) Infants with Death: 5 (IVB group) 2 (LPC group)	Low overall risk of bias (“Low” in RoB 2.0 tool)

*anti-VEGF* anti-vascular endothelial growth factor, *LPC* laser photocoagulation, *TR-ROP* treatment-requiring retinopathy of prematurity, *RoB 2*: version 2 of the Cochrane risk-of-bias tool for randomized trials, *ROBINS-I*: risk of bias in non-randomized studies of interventions, *ROP* retinopathy of prematurity, *IVB* intravitreal bevacizumab, *RD* retinal detachment, *APROP* aggressive posterior retinopathy of prematurity, *IVR* intravitreal ranibizumab, *AE* adverse event, *CA* corrected age, *PMA* postmenstrual age, *PTA* post term age

### Disease recurrence/disease regression/need for retreatment

In the RAINBOW study [6], treatment success, defined as alive and without treatment switch and unfavorable structural outcome or active ROP at day 169, was reported as odds ratio (OR) and 95% Confidence Interval (CI) in pairwise comparisons of the treatment arms: OR = 2.19 (CI 0.99 to 4.82; *P* = 0.050) of 0.2 mg.

IVR compared to LPC, and OR = 1.57 (95% CI: 0.76 to 3.26) of 0.1 mg IVR compared to LPC. This outcome is at low overall risk of bias.

The results of all the other RCTs (except RAINBOW), and the observational studies, that compared the rates of disease recurrence, disease regression, need for retreatment between IVB, IVR and LPC groups, are displayed in Table 4 [5, 6, 10, 13, 19, 22, 29, 33, 36–40, 42, 44–46, 48–51].

**Table 4:** Randomized clinical trials and observational studies that compare disease recurrence, disease regression and need for retreatment between intravitreal anti-VEGF and LPC treatment modalities for TR-ROP

Authors/first author	Study Type	Year	Participants	Treatment modalities being compared	Outcomes	Risk-of-bias assessment with RoB 2 tool^8^ or with ROBINS-I tool^9^
Kang et al. [10]	Observational study	2019	52 eyes of 27 infants with TR-ROP	IVB (0.625 mg/0.025 ml), IVR (0.2 mg/0.02 ml), Diode LPC	No differences between groups in: ROP recurrence requiring retreatment (*P* = 0.120)	Serious overall risk of bias due to bias in measuring of the outcome
Roohipoor et al. [13]	Randomized clinical trial	2019	232 eyes of 116 infants with type 1 ROP in zone II (stage 2 or 3 ROP with plus disease)	IVB (0.625 mg/0.025 ml), Diode LPC	No differences between groups in: Rate of complete vascularization of non-ablated retina and ROP regression at 90 weeks PMA (*P* = 0.200)	High overall risk of bias (“High” in the RoB 2 tool)
Lyu et al. [42]	Observational study	2019	27 eyes of 14 infants with type 1 ROP	IVR (0.25 mg/0.025 ml), Diode LPC	IVR group had: Higher rate of eyes with additional treatment due to ROP resistance, ROP reactivation, ROP progression and due to avascularity in zone 1 or zone 2 (*P* = 0.040) No differences between groups at: Rates of regression of acute ROP after primary treatment (*P* = 0.330)	Serious overall risk of bias due to baseline confounding
Zhang et al. [44]	Observational study	2019	283 infants with TR-ROP	IVB (dose unspecified)**,** LPC (type unspecified)	Anti-VEGF group had: Greater odds of having a second procedure (*P* < 0.001)	Serious overall risk of bias due to bias in measurement of the outcome
Kang et al. [36]	Observational study	2019	314 eyes of 165 infants with type 1 ROP	IVR (0.25 mg/0.025 ml), Diode LPC	Diode LPC group had: Higher rate of infants that underwent additional laser (*P* = 0.010) Higher rate of additional scleral encircling (scleral buckling (*P* = 0.040) No differences between groups at: Infants requiring addition IVR injection (*P* = 0.010) Need for vitrectomy (*P* = 0.660)	Serious overall risk of bias due to bias in the measurement of the outcomes
Barry et al. [46]	Observational study	2019	222 eyes of 115 infants with type 1 ROP	IVB (0.375 to 0.625 mg per eye), LPC (type unspecified)	No differences between groups in: Rate of retreatment within the period of 8 weeks (*P* = 0.140, Fisher’s exact test)	Serious overall risk of bias due to bias in measurement of the outcome
Ling et al. [48]	Observational study	2019	340 eyes of 176 infants with type 1 ROP	IVB (0.625 mg/0.025 ml), IVR (0.25 mg/0.025 ml), Diode LPC	At 75 weeks PMA, no differences between groups in: ROP recurrence rate (*P* = 0.050) Rate of need for vitrectomy (*P* = 0.270) Both anti-VEGF groups had: Higher mean interval of recurrence from initial treatment than the LPC group (*P* < 0.001) ROP recurrence at a later age compared to LPC group (*P* = 0.005)	Moderate overall risk of bias
Roohipoor et al. [19]	Observational study	2018	986 eyes of 493 infants with type 1 ROP	IVB (0.625 mg/0.025 ml), Diode LPC	IVB group had: Higher rate of disease activity (*P* = 0.040) Lower mean time to complete regression (*P* = 0.001) No differences between groups at: Rates of retreatment (*P* = 0.060) Time between treatment and retreatment (*P* = 0.270)	The two first outcomes are at serious overall risk of bias due to bias in measurement of the outcome. The next two are at moderate overall risk of bias
Lepore et al. [37]	Randomized clinical trial	2018	42 eyes of 21 infants with type 1 zone 1 ROP	IVB (0.5 mg/0.02 ml), Diode LPC	IVB group FA findings 4 years after treatment: All eyes had abnormalities at the periphery (shunts, vessel leakage, avascular area, tangles, abnormal vessel branching/abnormalities at the posterior pole, such as hyperfluorescent lesions and absence of foveal avascular zone LPC group FA findings 4 years after treatment: leakage (1 eye), tangles and shunts (3 eyes), macular abnormalities (3 eyes)	High overall risk of bias (“High” in the RoB 2 tool)
Blair et al. [40]	Observational study	2018	36 eyes of 19 patients with APROP	IVB (0.5–0.625 mg /0.02–0.025 cc), Diode LPC	No differences between groups at: Rate of eyes with acute reactivation requiring retreatment (*P* > 0.050) Mean age of the treatment-requiring recurrence (*P* = 0.080)	Serious overall risk of bias due to baseline and time-varying confounding
Zhang et al. [39]	Randomized clinical trial	2017	100 eyes of 50 infants with Zone 2 TR-ROP (i.e., zone 2 stage 2 or 3 ROP with plus disease)	IVR (0.3 mg/0.03 ml), Diode LPC	The IVR group had: Greater rates of ROP recurrence (*P* = 0.001)	Medium overall risk of bias (“Some Concerns” in the RoB 2.0 tool)
Sukgen et al. [45]	Observational study	2017	31 eyes in 16 patients with stage 4A ROP	IVR (0.25 mg/0.025 ml), Diode LPC	No differences between groups at: Rates of requirement for vitreoretinal surgery due to ROP progression (*P* = 0.230)	Moderate overall risk of bias
Toy et al. [51]	Observational study	2016	58 eyes of 30 patients with type 1 ROP	IVB (0.625 mg/0.025 ml), Diode LPC	Rate of recurrence or angiographic demonstration of ischemia, leakage, or both requiring rescue laser was 91% in IVB group vs 0% in diode LPC	Serious overall risk of bias
Nicoară et al. [50]	Observational study	2016	46 eyes of 23 infants with APROP	IVB (0.625 mg/0.025 ml), Diode LPC	IVB group had: Greater rates of APROP regression (*P* < 0.001, McNemar test)	Moderate overall risk of bias
Karkhaneh et al. [38]	Randomized clinical trial	2016	158 eyes of 79 infants with zone 2/stage 2 or 3 ROP	IVB (0.625 mg/0.025 ml), Diode LPC	IVB group had: Higher rate of stage 3 ROP recurrence and retreatment (*P* = 0.020) No differences between groups in: Need for PPV (*P* = 0.540) Time between treatment and retreatment (*P* = 0.290)	Medium overall risk of bias (“Some Concerns” in the RoB 2.0 tool)
Kong et al. [22]	Observational study	2015	80 eyes of 42 patients with type 1 ROP	IVB (0.625 mg/0.025 ml), LPC (type unspecified)	No differences between groups at: Rate of retreated eyes (*P* = 0.330)	Serious overall risk of bias due to baseline confounding
Hwang et al. [29]	Observational study	2015	54 eyes of 28 infants with type 1 ROP	IVB (0.625 mg/0.025 ml), Diode LPC	No differences between groups at: Rates of ROP recurrence (*P* = 1.000)	Moderate overall risk of bias
Warren et al. [33]	Observational study	2015	47 infants with threshold ROP	IVB (dose unspecified), LPC (type unspecified)	Anti-VEGF group had: Longer time to maturation (*P* < 0.050) No differences between groups at: Rates of patients requiring retreatment (*P* > 0.050) Patients requiring vitrectomy (*P* > 0.050)	Serious overall risk of bias due to baseline and time-varying confounding
Moran et al. [49]	Randomized clinical trial	2014	28 eyes of 14 infants with symmetrical zone 1 or posterior zone 2 Stage 3+ ROP	IVB (1.25 mg/0.1 ml), Diode LPC	Recurrence requiring retreatment: 3 eyes (21.42%) of the IVB group, 1eye (7.14%) of the diode LPC group IVB group had Longer time until recurrence (measured in weeks PMA) (*P* < 0.050)	High overall risk of bias (“High” in the RoB 2 tool)
Mintz-Hittner et al. [5]	Randomized clinical trial	2011	300 eyes of 150 infants with ROP (zone 1 or zone 2 posterior stage 3+)	IVB (0.625 mg/0.025 ml), Diode LPC	IVB group had: Lower rates of ROP recurrence compared to the LPC group (OR = 0.17, 95% CI: 0.05–0.53; *P* = 0.002, for zones 1 and 2 combined) Other outcomes: Performance of PPV: 2 eyes (IVB group), 13 eyes (LPC group)	Medium overall risk of bias (“Some Concerns” in the RoB 2.0 tool)

*anti-VEGF* anti-vascular endothelial growth factor, *LPC* laser photocoagulation, *TR-ROP* treatment-requiring retinopathy of prematurity, *RoB 2* version 2 of the Cochrane risk-of-bias tool for randomized trials, *ROBINS-I* risk of bias in non-randomized studies of interventions, *ROP* retinopathy of prematurity, *IVB* intravitreal bevacizumab, *OR* odds ratio, *CI* confidence interval, *PPV* pars plana vitrectomy, *IVR* intravitreal ranibizumab, *PMA* postmenstrual age, *FA* fluorescent angiography, *APROP* aggressive posterior retinopathy of prematurity

### Neurodevelopmental outcomes

#### Observational studies

The observational studies [15, 17, 22, 36, 44, 47, 52–55] that compared neurodevelopmental outcomes between intravitreal anti-VEGF and LPC for TR-ROP were of moderate or serious overall risk of bias and found many similarities between the two treatment modalities, while in case a significant difference existed, LPC had the better results in terms of neurodevelopmental outcomes (Table 5).

**Table 5:** Observational studies comparing neurodevelopmental outcomes between intravitreal anti-VEGF and LPC treatment modalities for TR-ROP

Authors/first author	Year	Participants	Treatment modalities being compared	Outcomes	Risk of bias assessment with ROBINS-I tool^9^
Arima et al. [53]	2020	53 infants with type 1 ROP	IVB (0.625 mg/0.025 ml), LPC (type unspecified)	At 18 months CA, IVB group had: Lower mean score in the Language-Social domain DQ (*P* = 0.010), even after the adjustment for GA and birth weight (*P* = 0.030) No differences between groups in KSPD scores at 18 months CA, for: Postural-Movement domain DQ (*P* = 0.100) Cognitive-Adaptive domain DQ (*P* = 0.170) Overall domain DQ (*P* = 0.100)	Moderate overall risk of bias
Raghuram et al. [15]	2019	60 eyes of 34 infants with TR-ROP	IVB (0.625 mg/0.025 ml), Diode LPC	At 18–24 months CA, no differences between groups in rates of: Moderate to severe NDI (*P* = 0.380) Severe NDI (*P* = 0.120) Cerebral palsy (*P* = 0.500) Hearing loss requiring amplification (*P* = 0.690) Motor Bayley scores (*P* = 0.580) Cognitive Bayley scores (*P* = 0.830) Language Bayley scores (*P* = 0.450)	Moderate overall risk of bias
Therani et al. [17]	2019	111 eyes of 64 infants with TR-ROP	IVB (dose unspecified), LPC (type unspecified)	At 18–24 months CA, no differences between groups in: NDI scores (OR = 1.63; 95% CI: 0.54–4.87, adjusted OR = 1.77; 95% CI: 0.46–6.73) sNDI scores (OR = 2.19; 95% CI: 0.80–5.98, adjusted OR = 2.31; 95% CI: 0.75–7.14)	Serious overall risk of bias due to missing information in three domains of bias assessment
Kang et al. [36]	2019	314 eyes of 165 infants with type 1 ROP	IVR (0.25 mg/0.025 ml), Diode LPC	Mean follow-up: 36.3 ± 31.9 months. No differences between groups in: Rate of adverse neurodevelopmental outcomes (*P* = 0.73)	Moderate overall risk of bias
Zhang et al. [44]	2019	283 infants with TR-ROP	IVB (dose unspecified), LPC (type unspecified)	After 2 years of follow-up, no differences between groups in rates of: CP (*P* = 0.060, adjusting for IVH) Motor delay (*P* = 0.540) Cognitive delay (*P* = 0.680) Language delay (*P* = 0.060)	Serious overall risk of bias due to missing information in two domains of bias assessment
Chen et al. [54]	2018	49 eyes of 25 patients with TR-ROP	IVB (0.625 mg/0.025 ml), Diode LPC	At an average of 20 months CA, no differences between groups in: Rates of neurodevelopmental delay at 20 months CA (adjusted OR = 0.87; 95% CI, 0.08–9.46)	Moderate overall risk of bias
Peyton et al. [47]	2018	22 infants with type 1 ROP	IVB (dose unspecified), LPC (type unspecified)	LPC group had: Higher rate of CP (*P* = 0.005) No differences between groups in Bayley-III standard scores for: Cognitive outcomes (*P* > 0.050) Language outcomes (*P* > 0.050) Motor outcomes (*P* > 0.050) Rate of neurodevelopmental delay (*P* = 0.090) Rate of hearing loss (*P* = 0.770) Rate of bilateral vision loss (*P* = 0.100)	Serious overall risk of bias due to baseline confounding
Lien et al. [52]	2016	62 infants with type 1 ROP	IVB (0.625 mg/0.025 ml), Diode LPC, Combination of IVB (0.625 mg/0.025 ml) and diode LPC	24 months after treatment diode LPC group had: Higher mean MDI score (*P* = 0.030) Higher mean PDI score (*P* = 0.002) than the IVB + LPC group No differences between three groups in: MDI and PDI scores measured at 6 months, 12 months, and 18 months after treatment (*P* = 0.380 and 0.830, 0.790 and 0.056, 0.100 and 0.697, respectively)	Moderate overall risk of bias
Morin et al. [55]	2016	125 infants with TR-ROP	IVB (dose unspecified), LPC (type unspecified)	At 18 months CA, LPC group had: Higher motor composite score (*P* = 0.020) Lower rate of neurodevelopmental disabilities (OR = 3.1; 95% CI: 1.2–8.4) No differences between groups in: Language composite scores (*P* > 0.050) Cognitive scores (*P* > 0.050)	Moderate overall risk of bias
Kong et al. [22]	2015	80 eyes of 42 patients with type 1 ROP ||	IVB (0.625 mg/0.025 ml), LPC (type unspecified)	At 1 year CA, no differences between groups in: Gross motor DQ (*P* = 0.780) Visual motor DQ (*P* = 0.840) Language DQ (*P* = 0.840) Cognitive DQ (*P* = 0.830)	Serious overall risk of bias due to time-varying confounding

*anti-VEGF* anti-vascular endothelial growth factor, *LPC* laser photocoagulation, *TR-ROP* treatment-requiring retinopathy of prematurity, *ROBINS-I* risk of bias in non-randomized studies of interventions, *IVB* intravitreal bevacizumab, *CA* corrected age, *NDI* neurodevelopmental impairment, *OR* odds ratio, *CI* confidence interval, *sNDI* significant neurodevelopmental impairment, *ROP* retinopathy of prematurity, *IVR* intravitreal ranibizumab, *CP* cerebral palsy, *IVH* intraventricular hemorrhage, *MDI* mental developmental index, *PDI* psychomotor developmental index, *DQ* developmental quotient, *GA* gestational age, *KSPD* Kyoto scale of psychological development

In the RCT of Kennedy et al. [56], 16 infants of the BEAT-ROP study were evaluated for medical and neurodevelopmental outcomes at 18–28 months corrected age (CA). The authors reported similar results in all outcomes when comparing the two treatment groups at follow-up (median cognitive score *P* = 0.060, language score *P* = 0.180, motor composite score *P* = 0.220, gross motor function level *P* = 0.850, rate of cerebral palsy *P* = 1.000, median CA *P* = 0.100, median weight percentile for age *P* = 0.270, median length percentile for age at follow-up *P* = 0.390, median head circumference percentile for age at follow-up *P* = 0.460). These outcomes are at medium overall risk of bias. This is the only RCT that we found concerning the comparison of neurodevelopmental outcomes between intravitreal anti-VEGF and LPC treatment modalities for TR-ROP.

### OCT and OCTA measurements

The observational studies [16, 20], that compared the macular OCT and OCTA measurements between intravitreal anti-VEGF and LPC in infants with TR-ROP were of critical overall risk of bias and in case a significant difference existed, IVB treatment showed lower mean foveal, parafoveal, perifoveal, and inner foveal thickness (Table 6).

**Table 6:** Observational studies comparing OCT and OCTA measurements between intravitreal anti-VEGF and LPC treatment modalities, in infants with TR-ROP

Authors/first author	Year	Participants	Treatment modalities being compared	Outcomes	Risk of bias assessment with ROBINS-I tool^9^
Chen et al. [16]	2019	47 eyes of 25 infants with type 1 ROP	IVB (0.625 mg/0.025 ml), Diode LPC	At 1 year post-treatment, IVB group had: Lower mean IFT (*P* = 0.002) Lower mean foveal VD (*P* = 0.020) Higher mean FAZ (*P* = 0.004) Higher mean parafoveal VD (*P* = 0.010) No differences between groups in: Mean OFT (*P* = 0.180) Mean subfoveal CT (*P* = 0.450) Mean perifoveal VD (*P* = 0.460) Mean foveal VD (*P* = 0.050)	Critical overall risk of bias due to selection bias
Lee et al. [20]	2018	80 eyes of 42 patients with type 1 ROP	IVB (0.625 mg/0.025 ml), Diode LPC, IVB (0.625 mg/0.025 ml) + diode LPC	The IVB monotherapy group had: Lower mean foveal thickness (*P* < 0.010) Lower mean parafoveal (*P* < 0.010) Lower mean perifoveal thickness (*P* < 0.010) No differences between groups in: Subfoveal CT (*P* = 0.210) Macular CT (*P* = 0.230)	Critical overall risk of bias due to selection bias

*OCT* optical coherence tomography, *OCTA* optical coherence tomography angiography, *anti-VEGF* anti-vascular endothelial growth factor, *LPC* laser photocoagulation, *TR-ROP* treatment-requiring retinopathy of prematurity, *ROBINS-I* risk of bias in non-randomized studies of interventions, *ROP* retinopathy of prematurity, *IVB* intravitreal bevacizumab, *IFT* inner foveal thickness, *VD* vessel density, *FAZ* foveal avascular zone, *OFT* outer foveal thickness, *CT* choroidal thickness

Finally, studies that compared other reported outcomes [54, 57–59], such as serum-free VEGF levels, serum Insulin-like growth factor 1 (IGF-1) levels, tricuspid *E*-wave values, odds of returning to respiratory baseline by 48 h, number of diagnoses at time of discharge, hospitalization days, days for oxygen requirement, and duration of hospitalization, are summarized in Table 7.

**Table 7:** Studies comparing different reported outcomes between intravitreal anti-VEGF and LPC/PRP as treatment modalities for TR-ROP that are useful for clinical ophthalmologists

Authors/first author	Study type	Year	Participants	Treatment modalities being compared	Outcomes	Risk of bias assessment with RoB 2 tool^8^ or with ROBINS-I tool^9^
Cilsal and Sukgen [58]	Observational study	2020	51 infants with TR-ROP	IVA (1 mg/0.025 ml), Diode LPC, control group	IVA group had: Higher tricuspid *E*-wave values compared to the diode LPC group (*P* = 0.020) Control group had: Lower tricuspid *E*-wave values compared to the two treatment groups (*P* = 0.040)	Serious overall risk of bias due to baseline confounding
Barry et al. [59]	Observational study	2019	138 infants with TR-ROP	IVB (dose unspecified), PRP (type unspecified)	IVB group had: More odds of returning to their respiratory baseline by 48 h (OR: 0.18; 95% CI: 0.05–0.67)	Serious overall risk of bias due to missing information in two domains of risk of bias assessment
Chen et al. [54]	Observational study	2018	49 eyes of 25 patients (8 patients with Zone I plus with stage 1, 2, 3; no plus stage 3, 2 patients with Zone II no plus with stage 1, 2, 3, 14 patients with Zone II plus with stage 1, 2, 3, and one patient with Zone III plus) with treatment-warranted ROP	IVB (0.625 mg/0.025 ml), Diode LPC	IVB group had: Lower number of diagnoses at time of discharge (*P* = 0.004) No differences between groups in: Mean of hospitalization days (*P* = 0.110 for total length of stay and *P* = 0.680 for length of stay after primary treatment) Days for oxygen requirement (*P* = 0.910 for duration of hospitalization, *P* = 0.790 after initial treatment)	Serious overall risk of bias due to bias in measurement of the outcomes
Kong et al. [57]	Randomized clinical trial	2015	24 infants with type 1 ROP	IVB (0.625 mg per eye per dose), IVB (0.25 mg per eye per dose), Diode LPC	Both IVB groups had: More decreased serum-free VEGF levels than the LPC group (*P* < 0.001) More decreased serum IGF-1 levels than the LPC group (*P* < 0.050)	High overall risk of bias (“High” in RoB 2 Tool)

*anti-VEGF* anti-vascular endothelial growth factor, *LPC* laser photocoagulation, *PRP* panretinal photocoagulation, *TR-ROP* treatment-requiring retinopathy of prematurity, *RoB 2* version 2 of the Cochrane risk-of-bias tool for randomized trials, *ROBINS-I* risk of bias in non-randomized studies of interventions, *IVB* intravitreal bevacizumab, *ROP* retinopathy of prematurity, *IGF-1* insulin-like growth factor 1, *IVA* intravitreal aflibercept, *OR* odds ratio, *CI* confidence interval

## Discussion

This systematic review of the literature regarding treatment of ROP revealed that higher quality studies concluded that LPC leads to greater rates of myopia than intravitreal anti-VEGF treatment, while the rate of adverse events and of unfavorable neurodevelopmental outcomes is similar. However, there was controversy among the included studies concerning the rate of ROP recurrence. Studies agree on findings regarding the refractive outcome, the rates of adverse events, and the neurodevelopmental outcomes, while they differ in disease recurrence rates. Notably, authors defined their outcomes differently and results are thus not directly comparable. Furthermore, most studies are observational and of moderate risk of bias, so safe conclusions cannot be drawn.

The RCTs [14] and the observational studies [15, 19] of the highest quality concluded that eyes treated with LPC developed more myopic refraction than the eyes treated with intravitreal anti-VEGF injections, a finding shared with the majority of observational studies as well [16, 20–23, 27–29, 33–36]. Rates of adverse events, complications and unfavorable outcomes were similar between intravitreal anti-VEGF agents and LPC in high quality RCTs like RAINBOW [6] and BEAT-ROP [5]. Results in lower-quality RCTs [13, 37–39] and observational studies [10, 19, 22, 27, 31, 33, 36, 40–47] did not generally differ.

Disease recurrence, disease regression and need for retreatment were similar between intravitreal anti-VEGF injections and LPC in the RAINBOW study [6], the highest-quality RCT available in our systematic review concerning that outcome. Some of the lower-quality RCTs concluded that the rate of disease recurrence was greater in the intravitreal anti-VEGF injection group [37–39], while others like BEAT-ROP [5] concluded the exact opposite. Finally, other RCTs [13] agreed with the findings of RAINBOW [6]. Some of the observational studies of higher quality concluded that rates of ROP recurrence were greater in the intravitreal anti-VEGF injection group [19], while others reported similar ROP recurrence rates between the two treatment groups [29, 45, 48], and others concluded that rates of ROP regression were greater in eyes treated with intravitreal anti-VEGF injections [50].

The only RCT that conducted a comparison of neurodevelopmental outcomes between intravitreal anti-VEGF and LPC, which was at medium overall risk of bias, found no differences in the neurodevelopmental outcomes between the two treatment modalities [56]. Most of the observational studies of higher quality also report this result [15, 36, 52, 54]. However, some high-quality observational studies supported that the intravitreal anti-VEGF group had worse neurodevelopmental outcomes in the Language-Social domain Developmental Quotient (DQ) at 18 months CA [53], motor composite score at 18 months CA [55], or more neurodevelopmental disabilities [55]. This may be due to the fact that there is only one RCT comparing neurodevelopmental outcomes between intravitreal anti-VEGF and LPC, and due to the observational design of the other studies.

The studies that reported OCT and OCTA measurements were of very low quality due to critical overall risk of bias and generally concluded that foveal thickness was lower, while mean foveal avascular zone (FAZ) was higher in the IVB group [16, 20].

Finally, an RCT with high overall risk of bias that compared the serum levels of free VEGF and IGF-1 between IVB and LPC treatment groups found lower serum levels of these two biochemical markers in the IVB group [57]. The observational studies that reported different outcomes related to the wide spectrum of adverse events were of very low quality and therefore analyzing these studies is out of the scope of this systematic review [54, 58, 59].

Intravitreal anti-VEGF treatment leads to lower rates of myopia, having a similar rate of adverse events and unfavorable neurodevelopmental outcomes as LPC. Therefore, intravitreal anti-VEGF treatment seems to have the preferable outcomes overall. However, no safe conclusions can be drawn concerning the rates of disease recurrence. More primary studies need to be conducted to give a definite answer to which treatment modality has greater rates of disease recurrence and to verify the aforementioned findings.

### Strengths and weaknesses

This study captured the comparison of all the adverse events, complications, and unfavorable structural and functional outcomes between intravitreal anti-VEGF injections and LPC that have been reported in the literature concerning the treatment of TR-ROP. Due to its systematic nature, this study aimed to summarize all current knowledge in view of facilitating clinical decisions. The adverse event comparison was stratified in sections that correlated with clinical significance, distinguishing different clinical entities of interest. Finally, the critical appraisal of the included studies was thoroughly conducted by assessing the risk of bias of each outcome of each individual study. Two independent researchers conducted the risk-of-bias assessment to limit bias as much as possible.

On the other hand, it should be mentioned that there is high heterogeneity between the included studies due to the very wide spectrum of the outcomes of our interest. This is the reason for not performing a quantitative synthesis of the results (meta-analysis).

Furthermore, some reported outcomes have been defined in different ways from study to study. For example, in the result section of disease recurrence or regression and need for retreatment, RAINBOW study defined treatment success as: alive and without treatment switch and unfavorable structural outcome or active ROP at day 169 [6], while BEAT-ROP study defined treatment failure as: the recurrence of neovascularization in one or both eyes arising from the retinal vessels and requiring retreatment by 54 weeks’ postmenstrual age [5]. That is, an obstacle encountered in many other outcomes of interest and therefore the included studies were synthesized in a descriptive way to succeed in providing the reader with conclusions that reflect everyday clinical practice. Lastly, an endogenous limitation that is related to the pathophysiology of ROP is that it is not clear if ROP recurrence is an adverse event or a failure of the respective treatment modality; therefore, ROP recurrence and adverse events were analyzed in different result sections.

### Clinical implications

Almost all the included studies, and most importantly, the higher quality studies like RAINBOW [6], agreed that LPC treatment leads to greater refractive errors and greater rates of myopia than intravitreal anti-VEGF treatment modalities. Similarly, almost all included studies reported no differences in the rates of adverse events, complications, unfavorable structural outcomes, and unfavorable neurodevelopmental outcomes between LPC and anti-VEGF. Findings of different high-quality studies, in terms of ROP recurrence/regression and need for retreatment, are overall controversial. This may be partly due to different definitions of ROP recurrence in the different studies that investigated this outcome or due to the vague nature of the outcome itself, because some authors interpret ROP recurrence as an adverse event, while others as failure of the applied treatment.

Eyes treated with LPC for TR-ROP tend to have more myopic refraction than the eyes treated with intravitreal anti-VEGF injections, while the rates of adverse events, complications, unfavorable structural outcomes, and unfavorable neurodevelopmental outcomes between the two treatment modalities seem to be similar. The RAINBOW study is designed to follow up participants until the age of five years so hopefully more data will become available soon [6]. There is a need for more primary studies, and a consensus needs to be agreed upon concerning the definition of the outcomes of interest. This would help in lowering the heterogeneity of future systematic reviews and in providing clinical ophthalmologists with more precise and more high-quality evidence about the comparison of intravitreal anti-VEGF injections and LPC in the treatment of TR-ROP.
